# Addition of rituximab to CHOP-like chemotherapy in first line treatment of primary mediastinal B-cell lymphoma

**DOI:** 10.1186/s12885-017-3332-3

**Published:** 2017-05-22

**Authors:** K. Lisenko, G. Dingeldein, M. Cremer, M. Kriegsmann, A. D. Ho, M. Rieger, M. Witzens-Harig

**Affiliations:** 1 Department of Hematology, Oncology and Rheumatology, University Hospital Heidelberg, Im Neuenheimer Feld 410, 69120 Heidelberg, Germany; 2 Oncology Outpatient Center, Darmstadt, Germany; 3 Institute of Pathology, University Hospital Heidelberg, Heidelberg, Germany

**Keywords:** Primary mediastinal B-cell lymphoma (PMBCL), Rituximab, Cyclophosphamide, Doxorubicin, Vincristine and prednisone (CHOP), International prognostic index (IPI)

## Abstract

**Background:**

The addition of rituximab (R) to CHOP (cyclophosphamide, doxorubicin, vincristine and prednisone) -like therapy has improved survival in primary mediastinal B-cell lymphoma (PMBCL) patients. However, these results were obtained in young low risk patients and a reevaluation in an unselected patient cohort is warranted.

**Methods:**

In this study, we analyzed 80 PMBCL patients treated with a CHOP-based regimen with and without rituximab.

**Results:**

In the non-rituximab cohort 10-year progression free survival (PFS) was 67% and 10-year overall survival (OS) was 72% versus a PFS of 95% and a OS of 92% in the rituximab group, PFS *P* = 0.001, OS *P* = 0.023. A subgroup PFS analysis by international prognostic index (IPI) risk revealed that all risk groups benefit from addition of rituximab to induction chemotherapy. In addition, OS probability was higher in the group of non-low risk patients who were treated with rituximab compared to those patients who did not receive rituximab (*P* = 0.035). In multivariate analysis, only addition of rituximab to induction chemotherapy and reaching complete remission (CR) after first line therapy had a beneficial effect on both PFS and OS, whereas IPI, age, upfront high dose (HD) chemotherapy/autologous blood stem cell transplantation (ABSCT) and rituximab maintenance had no impact on survival.

**Conclusions:**

Our data demonstrate a survival benefit in unselected PMBCL patients treated with CHOP-like induction regimen and additional rituximab independently of the IPI risk score.

## Background

Primary mediastinal large B-cell lymphoma (PMBCL) represents a distinct entity of mature B-cell lymphomas in the WHO 2008 classification and comprises 6–12% of all diffuse large B-cell lymphomas (DLBCL) and 2–4% of all non-Hodgkin’s lymphomas [1, 2]. It is usually diagnosed during the third and fourth decade and is slightly more common in women than in men [3–5].

The optimum treatment strategy in PMBCL patients (choice of chemotherapy regimen and use of radiotherapy) has so far not been determined by randomized clinical trials. As shown by retrospective analyses, compared to other forms of DLBCL, PMBCL appears to have a high incidence of primary chemotherapy resistance (up to 30%) [6] and relapse (over 20% after attained complete remission (CR) [4] upon cyclophosphamide, doxorubicin, vincristine and prednisone (CHOP) treatment without rituximab and poor prognosis of primary refractory or progressive disease [3, 6]. In the rituximab (R) era the management strategies of PMBCL in clinical practice are largely supported by uncontrolled prospective studies [7–9] and two main treatment options are conceivable: 6 to 8 cycles of dose-adjusted etoposide, prednisone vincristine cyclophosphamide, hydroxydaunorubicin (EPOCH-) R [8] or 6 cycles R-CHOP with consolidative mediastinal radiation therapy (Mabthera International Trial, [9]). In clinical practice the choice of treatment is guided by the consideration of potential long-term toxicities of radiation therapy, ease of administration and less short-term toxicity of R-CHOP plus radiation therapy versus the high risk of myelotoxicity (severe neutropenia in 50% of cycles) and hospitalization for neutropenic fever (13% of cycles) following dose-adjusted EPOCH-R [8].

However, the optimal management of PMBCL is not fully established. As shown by a recent retrospective study of 63 PMBCL patients by Soumerai et al. a high primary induction failure rate (21%) with R-CHOP was observed in an unselected patient cohort [10] while excellent outcomes (overall response rate 90%) were demonstrated in the Mabthera International Trial upon R-CHOP treatment [9]. These difference in therapy response can possibly be attributed to patient selection with a favorable prognosis under study conditions (patients <60 years with age-adjusted International Prognostic Index (IPI) score of 0–1, [9] versus 27% high-intermediate and 6% high-risk patients by age-adjusted IPI, median age 37 years, ranging from 20 to 82, [10]) and suggest a reevaluation of R-CHOP therapy in PMBCL patients in unselected patient cohorts.

In the current study we therefore retrospectively analyzed the outcome (therapy response, progression free and overall survival) of 80 PMBCL patients treated with a CHOP-based regimen with and without rituximab. Moreover, the significance of rituximab maintenance therapy was evaluated with regard to progression free and overall survival.

## Methods

### Study design, patient selection and data matching

All patients (*N* = 80) with newly diagnosed PMBCL that were treated at our institution from March 1992 to August 2013 were included into this retrospective analysis (observational study). All patients had histologically confirmed PMBCL. Clinical characteristics, IPI, [11]), first line therapy (type of induction chemotherapy, radiation therapy, high dose chemotherapy [HD]/autologous blood stem cell transplantation [ABSCT]), response to therapy were collected and retrospectively analyzed. Patients were grouped and evaluated with regard to rituximab treatment (yes/no) during induction chemotherapy or rituximab maintenance therapy (yes/no). Patients that were treated from 1992 to 2001/2002 did not receive rituximab during induction therapy. From 2001/2002 until 2013 rituximab was routinely administered as a part of induction therapy. 25 of 80 analyzed patients who participated in a multicenter prospective randomized HD2002 trial (rituximab maintenance versus observation in CD20+ B-cell lymphomas) received rituximab maintenance therapy. These patients were considered in the current study and were compared to those who did not receive rituximab for maintenance as a part of a subgroup analysis. The current study was approved by the ethics committee University Hospital Heidelberg without an informed consent of the patients with regard to its retrospectivity. Research was carried out in compliance with the Helsinki Declaration.

### First line therapy

All patients received a CHOP-based therapy as induction regimen: CHO(E)P (cyclophosphamide 750 mg/m^2^, i. v., day 1; doxorubincin 50 mg/m^2^, i. v., day 1; vincristine 1.4 mg/m^2^, maximum of 2 mg, i. v., day 1, [etoposide 100 mg/m^2^, i. v., days 1–3], prednisone 100 mg, p. o., days 1–5), and Mega-CHOEP (cyclophosphamide, doxorubincin, vincristine, etoposide, prednisone as previously described [12]). Rituximab (375 mg/m^2^) was administered as part of induction therapy at day 0 of each cycle on a regular basis starting at 2001/2002. Indication for involved field radiation therapy was bulky and extra-nodal disease. Consolidation HD-BEAM (carmustine, cytarabine, melphalan) and ABSCT were performed in 16 patients based on investigators choice. If applicable, in some patients (HD2002 trial) rituximab (375 mg/m^2^) maintenance therapy was administered every 3 months for 2 years.

### Response assessment

Therapy response was evaluated by clinical examination and computed tomography scan of the involved lymph node regions according to standardized response criteria for non-Hodgkin lymphomas/PMBCL [13].

### Statistical analysis

Statistical analysis was performed with R (R Development Core Team, 2008). For descriptive statistics data are presented as absolute numbers and percentage and as median and range unless otherwise stated. For the comparison of categorical variables, Fisher’s Exact test in case of 2 × 2 contingency tables or its Freeman-Halton extension in case of 2 x > 2 contingency tables were used. To identify differences among groups in case of continuous variables, a two sided independent t-test was performed. Progression-free and overall survival (PFS, OS) were calculated and plotted using Kaplan-Meier survival analysis. To calculate the differences between the engraftment curves, a log-rank test was used. Age, IPI, rituximab induction (yes/no), upfront HD/ABSCT, remission post first line therapy (non- CR versus CR) and rituximab maintenance (yes/no) were considered as clinically relevant parameters with regard to PFS and OS and were included into multivariate analysis. Cox proportional hazard model (semiparametric, estimation of the hazard ration [HR], confidence interval [CI]), method Breslow was used for multivariate analysis. For both multivariate PFS and OS analysis, the case number was 76, 12 events were observed and 4 observations deleted due to missingness. A *P*-value <0.05 was considered statistically significant. In case of multiple testing a *P*-value <0.05/k (k = number of tests) was regarded as statistically significant (alpha adjustment in accordance with the Bonferroni correction).

## Results

### Patients’ characteristics

Overall 80 patients with PMBCL were evaluated. Rituximab was administered to 45 patients and 35 patients did not receive rituximab during induction chemotherapy. With regard to the overall cohort the male:female ratio was 0.7. The median age at diagnosis was 37 (range 18–68) years. At initial diagnosis B-symptoms were reported by 40 (50%) patients. Advanced stage disease (stage II and IV) was observed in 20 (25%) patients. Low and low-intermediate risk IPI was found in 50 (66%) and 13 (17%) patients, respectively. 12 (16%) and 1 (1%) patients had high-intermediate and high risk IPI, respectively. No statistically significant differences in base line variables were observed between both treatment groups. Patients’ characteristics are shown in Table 1.

**Table 1:** Patients’ characteristics at PMBCL diagnosis

	Overall cohort	Rituximab treatment	No Rituximab treatment	*P* value
Patient number, *N* (%)	80 (100)	45 (56)	35 (44)	/
Age at diagnosis, years	37 (18–64)	38 (19–64)	35 (18–58)	0.485
Gender, *N* (%)				0.360
Male	33 (41)	21 (47)	12 (34)	
Female	47 (59)	24 (53)	23 (66)	
B symptoms, *N* (%)	40 (50)	26 (58)	14 (40)	0.176
LDH elevated, *N* (%)^a^	58 (79)	33 (75)	25 (86)	0.841
LDH level at diagnosis, median U/l (range)	329 (145–1450)	327 (112–1277)	376 (145–1450)	0.439
Ann Arbor stage, *N* (%)				0.396
I	11 (14)	8 (18)	3 (8)	
II	49 (61)	26 (58)	23 (66)	
III	15 (19)	7 (15)	8 (23)	
IV	5 (6)	4 (9)	1 (3)	
Extranodal sites involved, *N* (%)				0.536
no	68 (85)	37 (82)	31 (89)	
yes	12 (15)	8 (18)	4 (11)	
ECOG, *N* (%)				0.344
0	58 (73)	33 (73)	25 (71)	
1	20 (25)	12 (27)	8 (23)	
2	2 (2)	0 (0)	2 (6)	
IPI^b^, *N* (%)				0.397
Low risk	50 (66)	32 (71)	18 (58)	
Low-intermediate risk	13 (17)	7 (16)	6 (19)	
High-intermediate risk	12 (16)	5 (11)	7 (23)	
High risk	1 (1)	1 (2)	0 (0)	

^a^LDH was not available in 1 and 6 patients in the rituximab treatment and no rituximab treatment group, respectively. ^b^IPI was not available in 4 patients in the no rituximab treatment group. ECOG, Eastern Cooperative Oncology Group; IPI, International Prognostic Index; LDH, lactate dehydrogenase

### Significance of rituximab in induction therapy

First line treatment and response to therapy are summarized in Table 2 for patients treated with and without rituximab.

**Table 2:** PMBCL first line therapy

	Overall cohort	Rituximab treatment	No Rituximab treatment
Patient number, *N* (%)	80 (100)	45 (56)	35 (44)
Induction chemotherapy regimens, *N* (%)^a^			
6xR-CHOP	21 (27)	21 (47)	/
6xR-CHOEP	23 (29)	23 (51)	/
3xR-CHOP/3xR-CHOEP	1 (1)	1 (2)	/
CHOP, median 5 (range 1–8)	13 (16)	/	13 (38)
CHOEP, median 6 (range 3–7)	13 (16)	/	13 (38)
Mega-CHOEP, median 4 (range 1–5)	8 (10)	/	8 (24)
Response to induction chemotherapy, *N* (%)			
CR	17 (21)	6 (13)	13 (37)
PR	60 (75)	39 (87)	19 (54)
PD	3 (4)	0 (0)	3 (9)
Consolidation HD and ABSCT, *N* (%) upon:			
Mega-CHOEP	6 (8)	0 (0)	6 (17)
HD-BEAM	18 (23)	7 (16)	11 (31)
Overall	24 (30)	7 (16)	17 (49)
Radiation therapy, *N* (%)	70 (88)	41 (91)	29 (83)
Overall response after fist line therapy, *N* (%)^b^			
CR	32 (41)	15 (34)	17 (49)
PR	43 (54)	28 (64)	15 (43)
PD	4 (5)	1 (2)	3 (9)
Rituximab maintenance therapy, *N* (%)	25 (31)	24 (53)	1 (3)

^a^The induction chemotherapy regimen was not available in 1 patient in non-rituximab treatment group. ^b^First line therapy includes induction chemotherapy, and when appropriate in first line high dose (HD) chemotherapy and ABSCT and radiation therapy. Response after fist line therapy was not available in 1 patient in the rituximab treatment group. ABSCT, autologous blood stem cell transplantation; BEAM, carmustine, etoposide, cytarabine, melphalan; CR, complete remission; HD, high dose; PD, progressive disease; PR, partial remission; (R)-CHO(E)P, (rituximab), cyclophosphamide, hydroxydaunorubicin, vincristine, (etoposide), prednisone

**Fig. 1:**
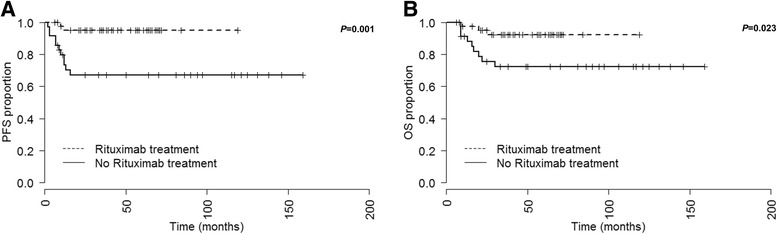
Progression-free survival (PFS, **a**) and overall survival (OS, **b**) of all patients treated either with (*N* = 45) or without (*N* = 35) rituximab. Median PFS and OS not reached

In the rituximab cohort the patients received either 6 cycles of R-CHOP or R-CHOEP as induction chemotherapy. 1 patient received 3 cycles R-CHOEP followed by 3 cycles R-CHOP. Upon induction chemotherapy 6 (13%) and 39 (87%) patients reached a CR and partial remission (PR) respectively and no primary induction failures were observed. 7 patients (16%, all of these patients reached PR after induction chemotherapy) underwent HD-BEAM chemotherapy and ABSCT. In none of these patients an improvement of remission status was observed upon HD chemotherapy and ABSCT (PR pre and post HD chemotherapy in all transplanted patients). 41 (91%) patients received radiation therapy. Upon radiation therapy an improvement of remission status from PR to CR was observed in 9 patients. In one patient the remission status was not available after radiation therapy and one patient developed progressive disease (PD) during radiation therapy. An overall response rate (ORR, CR and PR) of 98% (*N* = 43) was observed upon first line therapy. After a median follow-up of 42 (range 6–119) months relapse was reported in one patient. Projected 10-year PFS rate was 95% (Fig. 1a). 3 (7%) deaths occurred in the rituximab treatment group. Projected 10-year OS rate was 92% (Fig. 1b).

In the non-rituximab cohort 13 (38%) of patients received a median of 5 cycles of CHOP (range 1–8) or 6 cycles of CHOEP (range 3–7), respectively. 4 patients received 1–2 cycles of HAM post CHOP therapy. 8 (24%) of patients were treated with a median of 4 cycles Mega-CHOEP (range 1–5). One patient who was treated with 1 cycle Mega-CHOEP received 4 additional cycles CHOEP. In one patient the induction therapy regimen was not available. Upon induction chemotherapy CR and PR were reached in 13 (37%) and 19 (54%) patients, respectively. Primary induction failure was observed in 3 (9%) patients. Consolidation HD-BEAM or Mega-CHOEP and ABSCT were performed in 17 (49%) patients. Radiation therapy was performed in 29 (83%) patients. No changes in remission status were documented upon radiation therapy. In 1 patient a CR was reached upon surgical and radiation therapy. An ORR (CR and PR) of 91% (*N* = 32) was observed upon first line therapy. 1 patient received rituximab maintenance therapy within the HD2002 trial. The median follow-up time was 50 (2–159) months in the non-rituximab group. 7 patients relapsed after attaining PR after first line therapy. One patient relapsed after reaching CR with first line therapy. Overall PD/relapse was found in 11 (31%) patients in the non-rituximab cohort. Projected 10-year PFS rate was 67% (Fig. 1a). 9 (26%) death occurred in the non-rituximab treatment group. Projected 10-year OS rate were 72% (Fig. 1b).

**Fig. 2:**
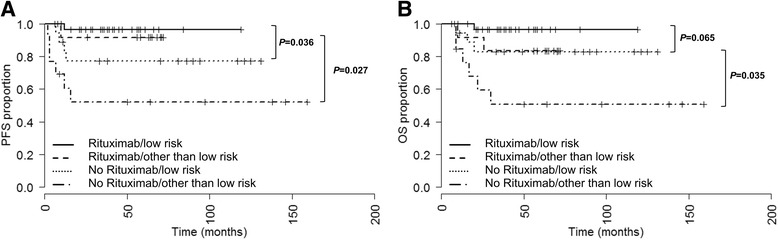
Progression-free survival (PFS, **a**) and overall survival (OS, **b**) of low and other than low (low-, high-intermediate, and high) risk patients by IPI treated either with (*N* = 32 and *N* = 13) or without (*N* = 18 and *N* = 13) rituximab. IPI was not available in 4 patients in the no rituximab treatment group. These patients were excluded from the survival analyses. Median PFS and OS not reached. Due to multiple testing (k = 2) a *P*-value <0.025 is considered as statistically significant

The PFS probability was found to be significantly higher in the rituximab treatment group compared to the non-rituximab cohort (*P* = 0.001, HR = 0.123, CI_95_ 0.041–0.374, Fig. 1a). A subgroup PFS analysis by IPI risk (low versus low-, high-intermediate, and high) revealed that both low risk and other than low risk PMBCL patients benefit from addition of rituximab to induction chemotherapy. However this result is limited by a low case number in each subgroup and showed a borderline significance: low risk subgroup *P* = 0.036, HR = 7.308, CI_95_ 1.168–45.703; other than low risk subgroup *P* = 0.027, HR = 7.442, CI_95_ 1.677–33.028, importantly due to multiple testing (k = 2) a *P*-value <0.025 is considered as statistically significant, Fig. 2a.

In patients who received rituximab during induction chemotherapy a significantly higher OS probability was observed (*P* = 0.023, HR = 0.248, CI_95_ 0.079–0.778, Fig. 1b). No statistical significance was observed in low IPI risk subgroups with regard to OS between those patients who received rituximab and those who did not (*P* = 0.065, Fig. 2b). In other than low IPI risk patients the OS probability was higher when rituximab was administered during induction therapy (*P* = 0.035, HR = 6.909, CI_95_ 1.564–30.511, importantly due to multiple testing (k = 2) a *P*-value <0.025 is considered as statistically significant, Fig. 2b).

On multivariate analysis addition of rituximab to induction chemotherapy and reaching CR after first line therapy (induction chemotherapy, and when appropriate HD chemotherapy/ABSCT and radiation) had a beneficial effect on both PFS and OS. No statistically significant impact of age or IPI score was observed (Table 3).

**Table 3:** Multivariate Cox proportional hazard analysis

	PFS		OS	
	HR (CI_95_)	*P*	HR (CI_95_)	*P*
Age	1.057 (0.984–1.135)	0.130	1.049 (0.981–1.121)	0.161
IPI	1.433 (0.365–5.620)	0.606	2.256 (0.580–8.767)	0.240
Rituximab induction	0.065 (0.011–0.367)	0.002	0.192 (0.041–0.890)	0.035
Upfront HD/ABSCT	0.318 (0.065–1.562)	0.158	0.498 (0.095–2.617)	0.410
Remission post first line therapy (non-CR versus CR)	0.106 (0.021–0.532)	0.006	0.156 (0.032–0.748)	0.020
Rituximab maintenance	0.444 (0.046–4.264)	0.482	0.360 (0.039–3.356)	0.370

*n* = 76; *ABSCT* autologous blood stem cell transplantation, *CI* confidence interval, *CR* complete remission, *HD* high dose, *HR* Hazard ratio, *IPI* International Prognostic Index, *OS* overall survival, *PFS* progression free survival

### Significance of HD chemotherapy and ABSCT

**Fig. 3:**
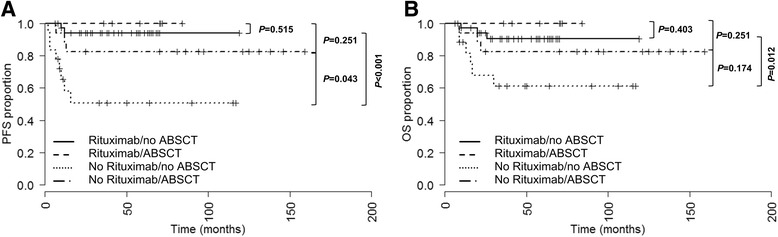
Progression-free survival (PFS, **a**) and overall survival (OS, **b**) of patients treated with and without consolidation high dose chemotherapy and ABSCT in dependency of rituximab treatment (with rituximab *N* = 7 and *N* = 38; without rituximab *N* = 17 and *N* = 18). Due to multiple testing (k = 4) a *P*-value <0.013 is considered as statistically significant

7 (16%) and 17 (49%) patients treated with and without rituximab received consolidation HD chemotherapy and ABSCT. In the rituximab cohort HD chemotherapy and ABSCT had no significant influence on PFS (*P* = 0.515, Fig. 3a) and OS (*P* = 0.403, Fig. 3b). In not rituximab treated patients a favorable outcome was observed upon HD chemotherapy and ABSCT with regard to PFS (*P* = 0.043, HR = 0.284, CI95 0.086–0.936, *P*-value of statistical significance <0.013, Fig. 3a) and OS (*P* = 0.174, Fig. 3b). However these results were not statistically significant. In patients who received HD chemotherapy and ABSCT rituximab treatment had no significant influence on PFS (*P* = 0.251, Fig. 3 a) and OS (*P* = 0.251, Fig. 3b). In contrast, in not transplanted patients rituximab during induction therapy significantly prolonged PFS (*P* < 0.001, HR = 11.165, CI95 0.2.735–45.568, *P*-value of statistical significance <0.013, Fig. 3a) and OS (*P* = 0.012, HR = 4.906, CI95 1.162–20.712, *P*-value of statistical significance <0.013, Fig. 3b). First line HD chemotherapy and ABSCT had no statistically significant impact on PFS or OS on mulitivariate analysis (Table 3).

### Significance of rituximab maintenance therapy

**Fig. 4:**
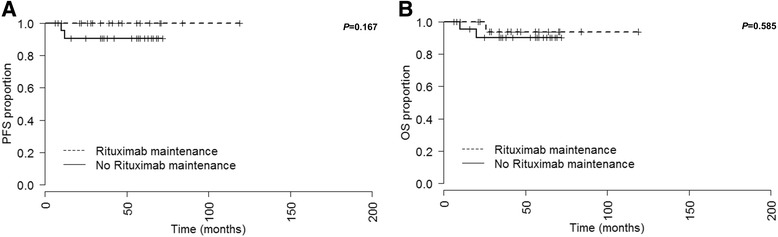
Progression-free survival (PFS, **a**) and overall survival (OS, **b**) of all patients that received rituximab during induction chemotherapy (*N* = 45) treated either with (*N* = 24) or without (*N* = 21) rituximab maintenance therapy. Median PFS and OS not reached

24 (53%) patients who had received a rituximab-containing induction therapy (overall *N* = 45) received rituximab maintenance therapy as a part of the multicenter prospective randomized HD2002 trial. Rituximab maintenance therapy (375 mg/m^2^) was administered for 2 years every 3 months. A PFS and OS evaluation and comparison to initially rituximab treated patients without rituximab maintenance therapy (*N* = 21, 47%) was performed for this patient cohort (Fig. 4). No statistically significant differences with regard to PFS (*P* = 0.167) and OS (*P* = 0.585) were observed between patients who received rituximab maintenance and those who did not. Rituximab maintenance therapy had no statistically significant impact on PFS or OS on mulitivariate analysis (Table 3).

We present a retrospective analysis of 80 patients with PMBCL treated with CHOP-like induction chemotherapy with or without rituximab. Our aim was to compare the therapeutic outcome between both treatments groups in a “real-life” population not selected for clinical trials. The limitations of this analysis can be attributed to its retrospectivety and the sequential evaluation of the patient cohorts with regard to the treatment periods without (1992 to 2001/2002) and with (2001/2002 to 2013) rituximab (possible influence of diagnostic and therapeutic innovations). However, the patient cohort was homogeneous with respect to clinical parameters at initial diagnosis (age, gender distribution, Ann Arbor stage, ECOG, IPI) among both treatment groups. The clinical variables corresponded well to the clinical PMBCL features described in the literature. In particular, PMBCL usually occurs during the third decade (rage of median age 27–42 years) and is more common in women than in men (46–78% of cases) [3, 5, 14–16]. In our patient cohort the median age was 37 years and 59% of patients were female. 50% of patients reported B-symptoms, which is slightly more compared to the patient cohorts described in the literature (24–48%) [6, 14, 17, 18]. Lactate dehydrogenase (LDH) elevation was found in 58% of patients, which is within the range reported by other authors (52–98% of cases) [6, 14, 19, 20]. About a quarter of patients have an advanced stage III or IV disease [4, 6, 15, 20]. In our patient cohort 19% and 6% of patients presented with stage III and IV PMBCL, respectively. Initial extranodal presentation is found in 10% to 69% of cases [14, 15] and was observed in 12% of cases in our study.

Upon CHOP-like induction chemotherapy without rituximab an ORR (CR 37%, PR 54%) of 91% was observed. 9% of patients showed primary induction failure and 23% of patients relapsed. These response rates correspond to the results obtained in retrospective multicenter analyses upon CHOP-like treatment without rituximab (CR 23%, 49%, and 51%; PR 41%, 32%, and 7%) [3, 4, 6]. However, the primary resistance to treatment observed in our patient cohort (9%) was lower compared to these multicenter studies (35%, 19%, and 42%) [3, 4, 6]. Lazzarino et al. reported an actuarial 3-year survival rate of 52% for a series of 99 PMBCL patients assessable for a CHOP-like induction regimen after a median follow-up of 50 months [6]. After a median follow-up from diagnosis of 52 months Zinzani et al. calculated projected 10-year PFS and OS rates for CHOP-like regimens of 35% and 44% respectively [4]. We observed higher 10-year PFS and OS rates (67% and 72%) in our cohort treated without rituximab.

As demonstrated by Rieger et al., rituximab significantly improved the outcome of PMBCL patients. Rituximab added to 6 cycles of CHOP-like chemotherapy increased the rates of complete remission (from 54% to 80% with ORR of 90%), virtually eliminated PD (2.5% versus 24%), and improved the 3-year event-free-survival (EFS, 78% versus 52%) compared to CHOP-like treatment only (Mabthera International Trial, [9]). Moreover, Dunleavy et al. reported excellent outcome results (event-free survival rate of 93% and OS rate of 97% during a median of 5 years of follow-up) upon rituximab and dose-adjusted EPOCH treatment without radiotherapy in previously untreated PMBCL patients [8]. However, these results were obtained on a preselected patient cohorts with favorable prognosis (patients <60 years [8, 9], age-adjusted IPI score of 0–1, extranodal involvement and stage III/IV PMBCL in 3% of patients [9]. On the contrary, a recent retrospective study of 63 PMBCL patients by Soumerai et al. demonstrated an unacceptably high rate of primary refractory disease on R-CHOP (21%), particularly among patients with high risk features (age > 60 years, 27% high-intermediate and 6% high-risk patients by age-adjusted IPI, advanced stage disease in 21% and mediastinal bulk in 71% of patients) [10]. The rituximab treated patient cohort evaluated in the present study closely corresponds to the patient group analyzed by Soumerai et al. (real-life situation, inclusion of patients over 60 years, advanced stage disease in 24% and not low risk IPI in 29% of cases). However, we could not confirm the poor therapy response retrospectively assessed by Soumerai et al. In this analysis, no patient treated with CHOP-like regimen and additional rituximab experienced primary resistance to induction therapy and CR and PR rates of 13% and 87% were reached. Although the CR rate was lower in patients who were treated with R-CHOP compared to CHOP therapy only (13% versus 37%), a favorable outcome with regard to PFS and OS was observed in patients that received rituximab (*P* = 0.001, and *P* = 0.023, respectively). In particular, in not HD chemotherapy/ABSCT setting rituximab during induction therapy significantly prolonged PFS and OS. Interestingly, consolidation with HD chemotherapy/ABSCT had no significant influence on PFS and OS when rituximab was administered during induction therapy. PMBCL patients treated with rituximab also had a beneficial PFS outcome when the cohort was stratified by IPI (low-, high-intermediate, and high). In higher, but not in low risk patients an advantageous OS outcome was observed upon rituximab treatment indicating the significance of rituximab addition to CHOP-like induction therapy in poor prognostic PMBCL groups. Overall, the addition of rituximab to induction chemotherapy and reaching CR after first line therapy had a beneficial effect on both PFS and OS on multivariate analysis.

Recently, rituximab maintenance therapy (375 mg/m^2^ every 3 months for 2 years) was shown to improve survival in male patients with DLBCL [21]. To the best of our knowledge, rituximab maintenance therapy has not been evaluated in PMBCL patients so far. In our analysis rituximab maintenance therapy did not seem to influence PFS and OS (*P* = 0.167, and *P* = 0.585, respectively). However, due to a low case number (*N* = 24) further evaluation is warranted in larger patient cohorts and prospective randomized trials.

## Conclusion

In conclusion, our data demonstrate an advantageous outcome (PFS and OS) in unselected PMBCL patients treated with CHOP-like induction regimen and additional rituximab over CHOP-like treatment only. Particularly in PMBCL patients with poor prognosis and those treated without consolidation HD chemotherapy and ABSCT rituximab containing induction therapy seems to provide a beneficial survival outcome.

## Abbreviations

ABSCT: autologous blood stem cell transplantation
BEAM: carmustine, cytarabine, melphalan
CHO(E)P: cyclophosphamide, doxorubicin, vincristine, etoposide, prednisone
CI: confidence interval
CR: complete remission
DLBCL: diffuse large B-cell lymphomas
ECOG: Eastern Cooperative Oncology Group
EFS: event-free-survival
EPOCH: etoposide, prednisone vincristine cyclophosphamide, hydroxydaunorubicin
HD: high dose
HR: hazard ration
IPI: international prognostic index
LDH: Lactate dehydrogenase
ORR: overall response rate
OS: overall survival
PD: progressive disease
PFS: progression free survival
PMBCL: primary mediastinal B-cell lymphoma
PR: partial remission
R: Rituximab
